# A Review of Multimodal Hallucinations: Categorization, Assessment, Theoretical Perspectives, and Clinical Recommendations

**DOI:** 10.1093/schbul/sbaa101

**Published:** 2020-08-09

**Authors:** Marcella Montagnese, Pantelis Leptourgos, Charles Fernyhough, Flavie Waters, Frank Larøi, Renaud Jardri, Simon McCarthy-Jones, Neil Thomas, Rob Dudley, John-Paul Taylor, Daniel Collerton, Prabitha Urwyler

**Affiliations:** 1 Neuroimaging Department, Institute of Psychiatry, Psychology and Neuroscience, Kings College London, London, UK; 2 Department of Psychiatry, Connecticut Mental Health Center, Yale University, New Haven, CT; 3 Department of Psychology, Durham University, Durham, UK; 4 School of Psychological Sciences, The University of Western Australia, Perth, Australia; 5 Department of Biological and Medical Psychology, Faculty of Psychology, University of Bergen, Bergen, Norway; 6 Psychology and Neuroscience of Cognition Research Unit, University of Liège, Liège, Belgium; 7 Norwegian Center of Excellence for Mental Disorders Research, University of Oslo, Oslo, Norway; 8 University of Lille, INSERM U1172, CHU Lille, Centre Lille Neuroscience and Cognition, Lille, France; 9 Laboratoire de Neurosciences Cognitives et Computationnelles, ENS, INSERM U960, PSL Research University, Paris, France; 10 Department of Psychiatry, Trinity College Dublin, Dublin, Ireland; 11 Centre for Mental Health, Swinburne University of Technology, Melbourne, Australia; 12 The Alfred Hospital, Melbourne, Australia; 13 Gateshead Early Intervention in Psychosis Service, Northumberland, Tyne and Wear NHS, Newcastle upon Tyne, UK; 14 School of Psychology, Newcastle University, Newcastle upon Tyne, UK; 15 Translational and Clinical Research Institute, Newcastle University, Newcastle upon Tyne, UK; 16 Gerontechnology and Rehabilitation, ARTORG Center for Biomedical Engineering, University of Bern, Bern, Switzerland; 17 Department of Neurology, University Neurorehabilitation Unit, University Hospital Bern—Inselspital, Bern, Switzerland

**Keywords:** hallucinations, multisensory, psychosis, computational

## Abstract

Hallucinations can occur in different sensory modalities, both simultaneously and serially in time. They have typically been studied in clinical populations as phenomena occurring in a single sensory modality. Hallucinatory experiences occurring in multiple sensory systems—multimodal hallucinations (MMHs)—are more prevalent than previously thought and may have greater adverse impact than unimodal ones, but they remain relatively underresearched. Here, we review and discuss: (1) the definition and categorization of both serial and simultaneous MMHs, (2) available assessment tools and how they can be improved, and (3) the explanatory power that current hallucination theories have for MMHs. Overall, we suggest that current models need to be updated or developed to account for MMHs and to inform research into the underlying processes of such hallucinatory phenomena. We make recommendations for future research and for clinical practice, including the need for service user involvement and for better assessment tools that can reliably measure MMHs and distinguish them from other related phenomena.

## Introduction

Various definitions have been advanced for “hallucinations,” but there is general consensus that a hallucination can be defined as a sensory experience that resembles veridical perception without having a corresponding sensory stimulation from the external environment.^1^ Hallucinations can occur in all senses, including auditory, visual, olfactory, kinesthetic, and more.^2^ Hallucinatory experiences span nosological categories^3^ and are a clinical manifestation of many psychiatric disorders (schizophrenia^4^ and bipolar^5^), neurodegenerative diseases (dementia with Lewy bodies [DLB]^6^), and Parkinson’s disease psychosis [PDP]^7^), as well as sensory disorders like hearing impairment or eye disease.^8,9^

Traditionally, hallucinations are often assumed to occur in one modality at a time (unimodal) and can be associated with different disorders—auditory hallucinations (AHs) in schizophrenia^10^ and visual hallucinations (VHs) in DLB.^11^ Where hallucinations do occur in different modalities, the predominant understanding is that they occur at different times (ie, they are not fused/simultaneous, like seeing and hearing a talking head; though see ^12^). Consequently, clinical assessments have had a focus on single modalities, thus biasing data collection toward unimodal hallucinations in potentially problematic ways. Nevertheless, growing recognition that hallucinations may occur in multiple modalities has shifted the attention to a systematic search for such multimodal phenomena.^3,6,13–17^

Despite the lack of in-depth scrutiny in the field, accounts of hallucinations across all senses can be traced through time.^18^ Historical examples include medieval descriptions of spiritual voice hearing, such as those by Margery Kempe,^19^ who did not just hear the “voice of God” but also had visions and other sensory experiences.^20^ A recent case study^21^ shows the experience of Mr T.A., a patient with schizophrenia who saw and heard humanoid creatures associated with a foul smell and who could go through his body, causing him unpleasant coenesthetic sensations (disorder of bodily perception^22^). Such examples challenge the notion that unimodal hallucinations are the overwhelmingly prevalent clinical manifestations of psychiatric and organic disorders^13–17,23^ and highlight the need for more accurate clinical assessment and management.

While hallucinations across multiple senses are starting to attract increasing research interest, several outstanding questions need to be addressed. This review, therefore, focuses on “multimodal hallucinations” (MMHs), ie, hallucinations that co-occur in different modalities, either in a simultaneous or in a sequential (serial) manner, with the overall objective of providing an overview of the field, highlight areas that require further scrutiny and identify issues of potential clinical importance.

## MMHs: Definition and Categorization

MMHs have been referenced in the literature by terms such as “polymodal/polysensory/intersensorial” and more,^24^ reflecting a lack of consensus on how to name, categorize, and understand such phenomena. There is confusion regarding MMHs at the level of a person’s range of experiences (an individual is prone to having MMHs) vs at the level of a hallucinatory episode (a particular experience can be classified as MMHs). If conceptualized at the person level, MMHs do not require temporal relatedness. Conceptualizing them at the level of a single hallucinatory episode would be more stringent but would also involve consideration of how closely together in time the hallucinations across modalities should occur to be considered part of the same hallucinatory episode (from simultaneously at some point to being in the same day or within the same psychotic episode).

Consequently, the lack of consistent specification regarding the temporal relationships that unimodal hallucinations might have with each other and whether they have to occur within a specific period to count as serial MMHs have made cross-comparisons of different studies difficult.

### Categorization

**Fig. 1.**
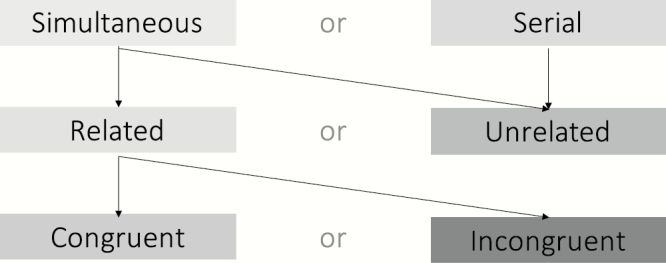
Categorization of multimodal hallucinations (level of a hallucinatory episode) across 3 dimensions: time, relatedness, and congruence. The arrows show how the different categories/levels can be combined to give rise to different types of multimodal hallucinations.

Given the lack of clarity in the literature, one aim of this review is to provide a categorization framework of MMHs along 3 dimensions (based on work in ^13,14,16^; figure 1. Examples of the different types of MMHs given by the possible combination of features along these dimensions can be found in table 1. For the implications of such framework for clinical and research practice see “Clinical Implications” section.

**Table 1.** Examples of multimodal hallucination (MMH) types based on the combinatorial features of MMHs along the 3 dimensions of time, relatedness, and congruence

Combinatorial features of MMHs						Example of MMH with such features
Serial	and	Unrelated			→	Seeing a dog today and hearing the voice of the devil a few days later
Simultaneous	and	Unrelated			→	Seeing a dog and hearing the voice of the devil
Simultaneous	and	Related	and	Congruent	→	Seeing a dog and hearing them bark
Simultaneous	and	Related	and	Incongruent	→	Seeing a dog and hearing them speak with the devil’s voice
Serial	and	Related	and	Congruent	→	Seeing a dog today and associating an auditory hallucination of barking noise later in the day to the same entity (ie, the dog)
Serial	and	Related	and	Incongruent	→	Seeing a dog today (visual hallucination only) and associating the voice of the devil heard later in the day (auditory hallucination only) to the same entity (ie, the dog)

The first dimension considers whether the hallucinations are occurring on the same temporal scale across multiple sensory modalities. If they co-occur, they are categorized as “simultaneous MMHs.” If they occur in one sensory modality at a time (unimodal) but in a sequential manner over time (with delays ranging from minutes, days, and more), they are categorized as “serial MMHs.” By conceptualizing such experiences at the person level instead, a clearer dimensionality emerges: one would consider the proportion of hallucinatory experiences that are in multiple modalities simultaneously vs in different modalities at different times.

The second dimension looks at whether the MMHs are experienced as being “from the same source or entity,” ^14,16^ but how to understand this concept of a *common source* is underspecified in the literature. A case study^25^ of a patient seeing and hearing the voice of a human figure shows how the common source can be based on perceptions of the same entity in different sensory modalities. However, hallucinations might also be of distinct but semantically related entities (eg, having a religious vision and subsequently hearing the voice of God). Importantly, considering relatedness in a dimensional rather than dichotomous way suggests 3 important points along this dimension: (1) cases of maximal relatedness in which MMHs represent the same entity; (2) cases of moderate relatedness in which MMHs represent distinct but meaningfully related entities; and (3) cases in which hallucinations across different modalities represent completely unrelated entities.

Finally, the third categorization level concerns whether the combination of hallucinations across modalities is contextually coherent (congruent MMHs) or not (incongruent MMHs). One limitation is that special combinations of the dimensions might give rise to definitional issues of MMHs. Would a (temporal) *sequence* of (conceptually) *unrelated* hallucinations occurring within a short time frame be considered multimodal? Perhaps yes.

It is important to highlight that this classification system is a clinical heuristic that would need further validation. Crucially, service users experiencing MMHs and unimodal hallucinations should be consulted in order to validate the current framework and available measures (discussed later) to ensure that one does not impose a classificatory system that limits their understanding of such experiences.

## Prevalence

Overall, studies show that hallucinations in one modality incrementally increase the risk of hallucinations in one or more other modalities.^26,27^ There also seems to be an inverse relationship between the number of modalities and proportion of people reporting them,^15,16^ as well as specific patterns of frequencies of MMHs across disorders, which will be discussed in the following sections.

### Schizophrenia and Bipolar Disorder

Since AHs were thought to be the cardinal symptom of psychotic disorders, other hallucinatory modalities were typically overlooked. Recent studies have, however, shown that, for schizophrenia, the weighted mean prevalence of VHs is around 27% (based on 29 studies)^6^ compared to 79% for AHs.^28^ Prevalence estimates for olfactory hallucinations vary from 6% to 26%, gustatory hallucination 1%–31% and somatic/tactile hallucinations 4%–19%.^15,28,29^

Evidence suggests that VHs in psychosis almost always (90% of cases) occur in combination with another hallucination modality (auditory, somatic, or other),^30,31^ in contrast to AHs, which can occur independently of other modalities about half of the time.^32^ The overall lifetime prevalence of any hallucinations for schizophrenia is approximately 80%, with MMHs being twice as common as unimodal ones (53% vs 27%).^16,33^ Such higher prevalence of MMHs over unimodal hallucinations was found across studies of bipolar disorder patients as well,^3^ suggesting continuity across psychotic illnesses. However, none of these studies specifically looked at simultaneous MMHs.

**Fig. 2.**
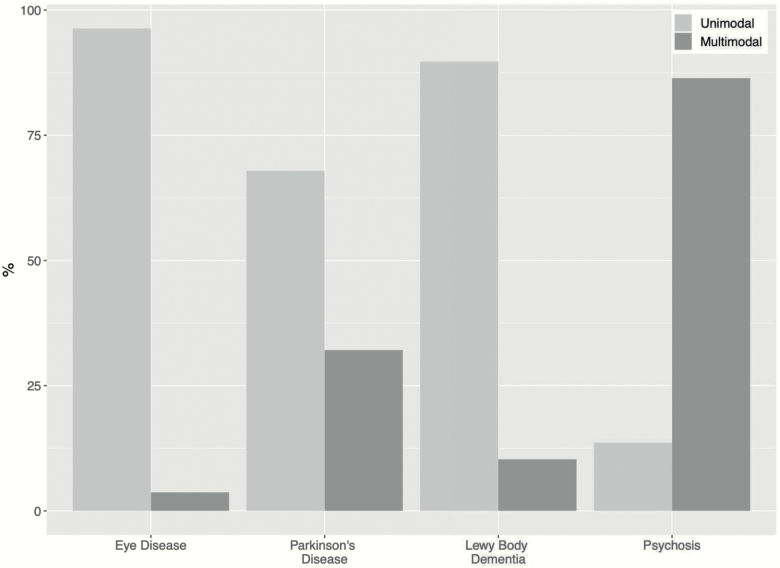
Bar chart showing the prevalence (in *n*%) of hallucination modalities in combination with visual hallucinations across 4 different disorders. Adapted from data in Table 2 in Dudley.^14^

Both serial and simultaneous MMHs were investigated in a group of 22 individuals with schizophrenia and VHs by Dudley et al.^13^ Ninety-six percent of patients experienced serial MMHs vs 86% experiencing simultaneous MMHs, indicating most had a combination of the 2. MMHs were again more common than unimodal ones (see figure 2). By contrast, others report that hallucinations in simultaneous multiple modalities are rare,^34^ suggesting that the relatively small sample in Dudley’s study^13^ might not necessarily represent the prevalence of simultaneous MMHs in psychosis more generally. Further replications are needed.

### Eye Disease and Neurodegenerative Disorders

Dudley et al^14^ compared hallucinations across different disorders in participants with VHs using the North East Visual Hallucination Interview (NEVHI)^35^ and found that the frequencies of unimodal vs multimodal experiences varied across neurodegenerative disorders (see figure 2). The Lewy Bodies Dementia (LBD) group had the highest prevalence of MMHs, followed by those with Parkinson’s disease (PD) and then by eye disease patients. Unimodal hallucinations remained the most prevalent for all groups, which is at odds with a study by Llorca.^30^ In their larger sample of 200 PD patients, a combination of hallucinations in 2–3 modalities was more common than unimodal ones, perhaps suggesting that bigger samples are needed to properly detect the prevalence of MMHs in neurodegenerative disorders.

In the Dudley study,^14^ despite the higher frequency of unimodal hallucinations reported by participants, MMHs were found to be more irritating, distressing, and frightening than their unimodal counterparts. Furthermore, patients’ VHs in the context of MMHs were accompanied by a significantly stronger degree of conviction about their veracity, suggesting some important clinical implications that need further exploration.

### Developmental Aspects

Hallucinations are frequently observed in children and adolescents in both clinical and nonclinical contexts.^36,37^ A phenomenological analysis of the hallucinations reported in the National Institute of Mental Health (NIMH) childhood-onset schizophrenia cohort revealed that the number of sensory modalities involved may serve as an indicator of the neurodevelopmental weight of the disorder.^26^ This idea of MMHs as a proxy of developmental vulnerability was also confirmed in a case series showing that the number of sensory modalities involved in early-onset hallucinations was related to the probability that a given child ever experienced prior traumatic events.^38^

## Assessment of MMHs

In order to better understand the phenomenology and prevalence of MMHs, it is important to have appropriate, valid, and reliable assessment tools that go beyond the measurement of unimodal experiences in the main sensory domains. To review the available measures fit for this purpose, published measures of MMHs across clinical and nonclinical populations were compared and summarized in table 2. For measures to be considered for review, they needed to include 3 or more modalities of hallucinations, be published, and used by researchers other than the developers and have evidence of their reliability and validity.

**Table 2.** Table showing the different scales for hallucinations alongside the sensory domains they assess and whether they capture if these hallucinations interact

Target population	Scale	Intent of the scale	AUD	VIS	OLF	S O M	Other	Assess temporal features (simul taneity)	Items n.	Features	Scoring and format	Format	Other
General population	LSHS (extended and modified versions)—Launay–Slade Hallucination Scale^49^	Hallucinations and related experiences							16	Presence	Items	Self-report	
	CAPS—Cardiff Anomalous Perception Scale^50^	Perceptual changes and hallucinations							32	Frequency, distress, and intrusiveness	Items	Self-report	
	MUSEQ—Multi-Modality Unusual Sensory Experiences Questionnaire^51^	Continuum perception— hallucinations							43	Frequency (+ distress, intrusiveness, worry, and impact)	Subscale	Self-report	
Children, adolescents, and youths	MHASC—Multisensory Hallucination Scale for Children^45^	Hallucinations							13/modality	Frequency, intensity, conviction, insight, control, discomfort, and emotional valence	Subscale	Both	App and online
	CAARMS—Comprehensive Assessment of At Risk Mental State^52^	Diagnostic interview							5	Frequency and duration	Items	Interview	
Psychotic disorders	SAPS—Scale for the Assessment of Positive Symptoms^53^	Broad symptoms profile							7	Frequency and severity	Subscale	Interview	
	DIP—Diagnostic Interview for Psychosis^54^	Diagnostic interview and symptoms							9	Presence and frequency	Items	Interview	
	KGV—Krawiecka, Goldberg, Vaughan psychosis scale^55^	Broad symptoms profile							6	Frequency, duration, subjective severity, and level of control	Items	Interview	
Across disorders	QPE—Questionnaire for Psychotic Experiences^56^	Hallucinations							11/50	Frequency, emotional content, distress, impact, and content	Subscale	Interview	Online
	NEVHI—North East Visual Hallucination Interview^35^	Hallucinations							21	Frequency, duration, severity, contents, emotional response, and behavioral response	Items	Self + carer interview	Semistructured
Parkinson’s disease and Alzheimer’s disease	UM-PDHQ—University of Miami Parkinson’s Disease hallucinations Q^57^	Broad symptoms profile (in Parkinson’s)							20	Frequency, duration, emotional response, and contents	Subscale	Self- report	
	MOUSEPAD—Manchester Oxford Universities Scale for the Psychopathological Assessment of Dementia^58^	Broad symptoms profile (in dementia)							14	Severity and duration	Items	Interview with carers	
	Rush Inventory^59^	Hallucinations (in Parkinson’s)							53	Frequency, contents, emotional response, duration, time of day, and context	Items	Self-report	
	TUHRAS—Tottori University Hallucinations Rating Scale^60^	Hallucinations (in Parkinson’s)							7	Frequency, severity, and vividness	Items	Interview	
	BEHAVE-AD—Behavioural Symptoms in Alzheimer’s Disease^57^	Broad symptoms profile (in Parkinson’s)							4	Clarity and behavioral response	Items	Interview	
	CUSPAD—Columbia University Scale for Psychopathology in AD^61^	Broad symptoms profile (in Alzheimer’s)							5	Presence and clarity	Items	Carer interview	

*Note:* AUD, auditory; VIS, visual; OLF, olfactory; SOM, somatosensory.

Overall, although several of the scales examined can be used to detect *serial* MMHs, only 4 out of 16 have items that specifically assess *simultaneous* MMHs, with most focusing on one modality (eg, visual domain in the NEVHI).

 To enable researchers and clinicians to reliably and validly detect both serial and simultaneous MMHs, scales need to include:

- assessments of the dimensional categories discussed;
- items detecting delirium (acute state characterized by attentional impairments, cognitive dysfunction, and fluctuating awareness of the surroundings^39^) as this can be common in neurodegenerative disease patients^40^ and their experiences might be mistaken for MMHs but be delirium episodes^41^;
- items assessing the presence of sleep disorders and incubus experiences in sleep paralysis as these can be linked to multisensory vivid experiences that might again be confused as MMHs (particularly in neurodegenerative disorders^42^;
- items evaluating experiences beyond the common 5 sensory modalities and what the relationship between them is.

## Theoretical Perspectives

Theories of hallucinations can be divided into those that consider these experiences to be attributable to modality-specific pathological processes and those that propose modality-general processes affecting multiple sensory modalities. An elegant discussion of these theories with regards to MMHs can be found in Fernyhough’s paper.^43^ The present review builds on this work and aims to synthesize how these theories stand up to scrutiny given the available evidence on MMHs.

A related question is to what extent the processes underlying MMHs are pathological. It could be that the pathology lies within one sensory system (visual in DLB or auditory in psychosis), and then the normal processes that ensure sensory consistency create MMHs from an initially unisensory experience (eg, you start to see something, priming you to later hear something consistent with it). This would be in line with an activation of modality-general representations, particularly of social agents, but, at present, we have little knowledge of how MMHs develop, both within a single episode and over time.

### Modality-Specific Processes

The fact that recent studies indicate that MMHs are more prevalent in psychiatric and organic disorders than previously assumed—albeit without a rigorous distinction between the exact type of MMHs—raises the question of how to best conceptualize these experiences across diagnostic categories and whether theories of unimodal hallucinations can account for such multimodality.

The presence of modality-specific processes of hallucinations is supported by evidence linking deficits in peripheral sensory systems to an increased likelihood of experiencing hallucinations in those domains. This is the case for VHs in eye diseases,^9^ which are associated with increasing visual impairments and abnormal activity of visual pathways. Analogously, in the auditory domain, Linszen et al^44^ found an association between hearing impairment and AHs, the latter worsening as a function of hearing loss severity. Capture studies in schizophrenia also showed that activity in the auditory cortex is linked to AHs.^45–48^ Finally, while not much research is available in other modalities, case studies in PD patients found an association between impaired sense of smell and the experience of olfactory hallucinations.^49^

Few brain imaging studies have explored the role of sensory complexity on the neural networks identified in hallucinations. The functional patterns associated with the occurrence of AHs, VHs, or auditory–visual hallucinations were investigated in an fMRI capture study on medication-free adolescents experiencing first-episode psychosis^50^ and confirmed the recruitment of physiological modality-specific pathways in these aberrant experiences. Furthermore, in schizophrenia,^51,52^ audio–visual hallucinations are associated with distinct functional and structural dysconnectivity patterns compared to those associated with unimodal AHs.

### Modality-General Processes

If both serial and simultaneous MMHs can be explained by the modality-specific processes discussed so far, one would expect to find sensory deficits across all sensory modalities in which patients experience hallucinations. To our knowledge, no empirical study has yet shown such a pattern of results, and the presence of MMHs across disorders cannot be fully explained by considering modality-specific processes alone, indicating the involvement of modality-general processes as well.

#### Misattribution Biases

One candidate process for explaining hallucinations across modalities is a general bias to misattribute internally generated representations (of any modality) to an externally generated source. This concept stems from the inner speech misattribution theory of AHs in schizophrenia,^53^ which posited that such experiences are the outcome of misattributing one’s inner speech to an external entity. As Fernyhough^43^ suggested, the same mechanism could be translated to other modalities, with evidence for its analog in vision: PD patients with VHs have stronger visual imagery than those without hallucinations.^54^ Furthermore, someone’s tendency to be a visualizer or verbalizer relates to their proneness to MMHs.^55^ Whether this stronger visual imagery is coupled with internal representations being unusually compelling—as is the case in the auditory domain—remains to be further investigated. It is unclear whether one can have internal smells or internal gustatory sensations equivalent to having internal speech. Further issues with this model’s explanatory power for MMHs are that, in its current form, it cannot explain the different rates of hallucinations across modalities in several disorders, as it would predict that all sensory systems will be equally affected, resulting in similar rates of hallucinatory experiences across them. Nevertheless, this discrepancy could be due to the fact that some sensations might not be equally salient across senses—as suggested earlier—or because some senses might have a different role/weight in perception in distinct scenarios. Thus, MMHs pose interesting challenges to misattribution bias theories that warrant further attention.

#### Reality Monitoring Deficit

Could MMHs reflect deficits in reality monitoring? This is the ability to discern internally from externally generated memories and such monitoring of the origin of information might go awry in some disorders.

Research suggests that reality monitoring relies on the anterior paracingulate sulcus (PCS) in the medial prefrontal cortex^56,57^ and evidence of its deficits in hallucinating patients is found across conditions (in schizophrenia^58,59^; in PD^60^). Work by Garrison et al^56^ showed a potential link between reality monitoring and MMHs. They found that, in a group of patients with schizophrenia, reduced length of the PCS was linked to a 19.9% increased likelihood of experiencing hallucinations and, crucially, this relationship was independent of hallucinatory modality (but see ^57,61^ for work on PCS in nonclinical voice hearers). Such evidence, coupled with the previous findings, suggests that reality monitoring might be a possible modality-general mechanism for explaining the emergence of MMHs.

Nevertheless, this theory still suffers from the same shortcomings discussed in previous models and it needs to be reconciled with the evidence of separable modality-specific reality monitoring systems^62^ that could be interacting with the modality-general ones. If so, it would be interesting to see if there are as many modality-specific reality monitoring systems as there are MMHs experienced by patients.

#### Social Agent Representations Theory

A third modality-general theory that considers the often-neglected social content of hallucinations is the “Aberrant Activation of Social Agent Representations” theory.^63^ Rather than specific sensory stimuli (voices/images), it is the general representation of another agent that is mistakenly activated, triggering the experience of that entity in all modalities. The importance of this theory for MMHs lies in its suitability for experimental testing: (1) one could investigate the extent to which different modalities relate to social agents and to what extent the relatedness dimension discussed earlier might correspond to this and (2) test the prediction that simultaneous and related MMHs should be more commonly experienced than other types. Currently, these hypotheses have not been tested, and further research is needed to explore the explanatory power of this theory for MMHs.

### Information Theory Frameworks: Predictive Coding and Circular Inference

The next set of theories cannot be easily characterized as either modality general or modality specific but have elements compatible with both. These theories share the view that hallucinations arise from a dysfunction in the interaction between top-down expectations and bottom-up information. They diverge in the specific way in which these sources of information are thought to interact.

The first theory is predictive coding (PC).^4,64^ It considers the mind as a hierarchical structure engaged in message passing in which perception is a generative process.^65^ The levels of this hierarchy are thought to mirror the hierarchical structure of the world at different levels of abstraction and what is passed along is a prediction error (PE),^66^ ie, the difference between what the higher level predicted about the input from the lower level and what the signal actually was.^67^ The brain aims to minimize PEs^68^ so that what is expected and what is experienced are congruent. If PEs are falsely generated/assigned too much precision, the brain would mistakenly update its model of the world, potentially leading to altered perceptions (at the lower level, ie, hallucinations) and altered beliefs (at the higher level, ie, delusions). Research in schizophrenia and PD suggests that hallucinations might be linked to overreliance on top-down influences on perception,^69–72^ possibly suggesting similar trans-diagnostic mechanisms of hallucinatory experiences.

Within this framework, one would predict that experiencing hallucinations in one modality might increase the prior expectation (top-down mechanism) of experiencing a congruent hallucination in other modalities. This is in line with Dudley’s findings,^13^ where 88% of their early psychosis patients experienced congruent MMHs. Nevertheless, current conceptualizations of PC do not explain the emergence of incongruent and/or unrelated serial or simultaneous MMHs, nor do they account for hallucinations that are not consonant with one’s general model of the world—as is the case with unimodal or serial unrelated MMHs in which one experiences disembodied voices.^13^ Furthermore, there is underspecification of whether PE and priors are modality specific or modality general or whether they can be a combination of the 2. More details regarding this would allow one to test specific empirical hypotheses regarding MMHs within this PC framework and thus pave the way for further research in the field.

A related theory in hallucinations research involves the concept of circular inference (CI).^73^ The core idea is that hallucinations and delusions can be understood in the framework of information theory and, specifically, of message passing in the form of belief propagation in a hierarchical neural network. If not tightly controlled, information propagated in both bottom-up and top-down directions can be amplified and reverberated through “loops”—generating reverberation errors. Bottom-up sensory evidence could be erroneously taken as top-down expectations, while top-down information could be fed back up and be mistaken for sensory information, leading to “*expecting what we see”* or “*seeing what we expect,”* respectively.^74^ This system is thought to be under the control of the glutamatergic and GABAergic balance and, when it becomes dysfunctional, it can lead to hallucinations and delusions^75^—as found in schizophrenia.^76^

Evidence linking CI and MMHs comes from work on synesthesia, a perceptual phenomenon whereby stimulation of one modality leads to experiences in another modality^77^ and which has been linked to disinhibited feedback from association cortex to sensory cortex.^78^ Ongoing research using simulations of drug-induced synesthesia^79^ has shown that reverberation of information descending the cortical hierarchy (descending loops) could induce audio–visual experiences in the absence of clear sensory input (cross-modal hallucinations) but also a contamination of one modality by another in the presence of a unimodal stimulus (synesthesia). These 2 features were retained as good candidates for modeling psychedelic-induced subjective experiences using probabilistic approaches. Both ascending and descending loops could account for variations in the phenomenology of psychotic (or pharmacologically induced) hallucinations: AHs with reduced illusions in the former and audio–visual hallucinations with synesthesia in the latter.

### Functional Systems Approach: A Bridge Between Modality-Specific and Modality-General Processes?

Finally, it is important to consider how MMHs can be conceptualized through dysconnectivity between elements of a functional system,^43,80^ a framework that might allow one to understand how modality-general and modality-specific processes interact. Evidence of aberrant connectivity of neural networks has been found in relation to AHs in schizophrenia^81^ and to VHs in PD.^82,83^ There is some work on resting state and network connectivity in patients experiencing MMHs in schizophrenia, but no evidence is available for other disorders. One could speculate a hypothesis whereby the observed heterogeneity of unimodal and MMHs within and across groups could be attributable to a common modality-general process that gives rise to different clinical phenomenological manifestations depending on what part of the network it affects, alongside potential, although perhaps not sufficient and necessary, pathology in modality-specific networks.

## Clinical Implications

The clinical understanding of hallucinations has primarily focused on the auditory modality (exemplified by the fact that most interventions are predominantly for AHs^84^), with limited considerations of other modalities or of MMHs.

Nevertheless, preliminary data indicate that MMHs are linked to higher levels of adverse mental health outcomes, being perceived as more distressing, frightening, and more veridical than unimodal hallucinations.^13,17^ For serial MMHs, related phenomena can make hallucinations appear to have the power to affect the person in different ways: for instance, tactile sensations or visions that are meaningfully connected to a disembodied voice can contribute to beliefs that a voice has power over the individual.^85^

One should consider whether multimodal experiences are connected temporally (eg, “Do you usually see them when they are talking? Do you see them without hearing them?”) because it would be of relevance in formulating how hallucinations impact on distress. In psychological interventions, clarifying the temporal sequencing of hallucinatory experiences in different modalities may inform how these episodes unfold over time. For instance, the beliefs that people hold about how different hallucinations are interconnected may be targets for cognitive therapy methods. Furthermore, assessing a person’s response to the first hallucinatory episode, and their potential expectation for related experiences to occur, may indicate points of intervention via alternative coping strategies.

Finally, we need to evaluate if MMHs lead to poorer outcomes in treatment trials and research the effectiveness of antipsychotic medication for MMHs compared to unimodal ones. This is for 2 reasons: first, whilst antipsychotic medications have a broad effect on psychotic symptoms, and no drugs specifically target hallucinations,^86^ it is not known whether medication may differentially affect MMHs vs unimodal experiences within subjects. This is an area for further research. Second, given the role that antipsychotics have in the management of patients’ potential distress caused by hallucinations (which is often what differentiates clinical from nonclinical cases), it is important to extend the investigation of such efficacy beyond unimodal experiences and to the distress experienced by those specifically with MMHs.

## Unanswered Questions and Recommendations

Given the paucity of systematic evidence regarding MMHs, there are many unanswered questions and avenues for further research. First, despite some preliminary data, it is not clear whether and how the base modality of hallucinations (ie, the most prominent and frequent) changes the prevalence of experiencing hallucinations in other modalities. Second, longitudinal studies are necessary to ascertain if MMHs change over time. Third, the frequency of unusual experiences (eg, someone only having an MMH once a month but consistent unimodal ones daily) is important in understanding multimodality.

It also remains unclear how many senses one should take into account, since the established 5 sensory domains might not capture the whole range of hallucinatory experiences. Blom^87^ describes 14 “senses” in which hallucinations have been reported, thus widening the range of sensory modalities involved. This raises the question of whether hallucinations in less well-known sensory domains are equally understood by the experiencers themselves and whether they can be easily conveyed to others. Arguably, there might be general difficulties in conveying nonverbal experiences to others in general, which would be a significant challenge in the assessment of MMHs.

In addition, despite the large body of literature on culture and unimodal hallucinations (eg, in schizophrenia^28,88,89^ and in the general population^90^), there is no systematic evidence of its specific influence on MMHs. The underreporting of MMHs^12^ could be partially due to their lack of emphasis in standard psychiatric assessments (which may, in turn, be due to an overemphasis on auditory verbal hallucinations in Western psychiatry).^91^ Research should look at transcultural data and compare MMHs across groups, especially where there is a strong element of spirituality. It would be interesting to investigate whether some subcultures have stronger expectations that spiritual entities will manifest themselves in particular modalities over others, eg, vision and auditory for “spiritual jaguars” in the Amazonian Wari’ shamans,^92^ and vision/auditory/tactile/olfactory components in the experience of Jinns in Islamic cultures.^93^

Furthermore, although an overview of multisensory integration (MI—the ability to integrate information from different sensory sources)^94^ is beyond the scope of this paper, it is important to consider MMHs in light of recent evidence on multisensory processing. MI involves several brain areas and networks, starting as early as in the superior colliculus.^95^ A growing body of evidence attests to: (1) MI problems in schizophrenia both for low-level stimuli^96^ and more complex ones,^97^ indicating issues with faulty “binding” of stimuli in time and/or space,^94^ and (2) a link between these issues and hallucinations.^98^ Similarly, PDP patients with hallucinations have problems with the integration of perceptual and attentional processing.^99,100^ Therefore, findings linking MI problems to hallucinations raise the question of whether the heterogeneity of MMHs could be traced to different areas underlying MI, thus giving rise to different types of MMHs. This could be a very important avenue for further research and warrants further attention.

Finally, most current theories are not able to explain (1) why the rates of MMHs across modalities vary within an individual and across patient groups, (2) why patients can show a combination of simultaneous MMHs, serial MMHs, and unimodal hallucinations, and (3) what might give rise to the relatedness and/or congruency of the content of both simultaneous and serial MMHs.

To conclude, in line with the recommendations of the 2017 International Consortium on Hallucination Research,^101^ this review shows the need to carry out a systematic investigation of MMHs. Overall, the evidence of the high prevalence and adverse prognostic outcomes of MMHs across disorders highlights the need to develop better assessment tools and theoretical models to systematically investigate these experiences and inform treatment strategies alongside the help of service users who experience MMHs and unimodal hallucinations.
